# Women With Ovarian Cancer and With Fertility Preservation: A Survival Analysis Using the Surveillance, Epidemiology, and End Results Database and Construction of Nomograms to Predict Cancer-Specific Survival

**DOI:** 10.3389/fonc.2022.860046

**Published:** 2022-04-11

**Authors:** Yue-min Hou, Hui Yu, Jia-tao Hao, Fang Feng, Rui-fang An

**Affiliations:** Department of Gynecology and Obstetrics, The First Affiliated Hospital of Xi’an Jiaotong University, Xi’an, China

**Keywords:** fertility preservation, ovarian cancer, chemotherapy, prognosis, SEER

## Abstract

**Objective:**

This study aimed to determine the risk and prognostic factors of ovarian cancer (OC) in women having fertility-sparing surgery, as well as survival outcomes of those with stage I epithelial ovarian cancer (EOC). We also determined the effect of chemotherapy in OC treatment and used multiple independent risk factors to establish a prognostic nomogram model for patients with stage I EOC.

**Patients and Methods:**

Individuals with OC and with fertility-sparing surgery (FSS) between 1998 and 2016 were identified in the SEER database. Univariate and multivariate logistic regression was performed to identify the distributions of patient characteristics according to chemotherapy. Cancer-specific survival (CSS) was assessed using Kaplan–Meier curves and log-rank tests. Univariate and multivariate Cox regression was conducted to determine the independent prognostic factors for CSS. Cox analysis was used to construct a nomogram model. The C-index and calibration plots showed the performance evaluation results.

**Results:**

A total of 1,839 women with OC with FSS were identified in the SEER database. Factors associated with significantly higher odds of undergoing chemotherapy included younger age, being unmarried, having grades 2–4, stages II–III, or clear cell and non-epithelial histologic type following a multivariate logistic regression analysis. Multivariate Cox regression analysis confirmed that age, marital status, chemotherapy, histologic type, grade, and the International Federation of Gynecology and Obstetrics (FIGO) stage were independent prognostic factors for CSS. In stage I EOC, the prognosis in patients with stage IA/IB-grade 3 (5-year CSS 85.3%) or stage IC (5-year CSS 80.6%) was worse than that in those with stage IA/IB-grade 1 (5-year CSS 95.2%), or stage IA/IB-grade 2 (5-year CSS 94.7%). However, chemotherapy improved the survival of patients with stage IA/IB-grade 3 (5-year CSS 78.1% vs. 94.6%, *p* = 0.024) or stage IC (5-year CSS 75.1% vs. 86.7%, *p* = 0.170).

**Discussion:**

The study provided population-based estimates of risk factors and prognoses in patients with OC and with FSS as well as the survival outcomes of patients with stage I EOC and the effect of chemotherapy. The constructed nomograms exhibited superior prognostic discrimination and survival prediction for patients with stage I EOC.

## Introduction

Ovarian cancer (OC) is one of the most common gynecological malignancies and ranks as the fifth cause of death from cancer among women in the United States. Epithelial ovarian cancer (EOC) is most commonly diagnosed among women of post-menopausal age (1). According to the latest cancer statistics in the United States in 2021, 13,770 individuals died of OC and 21,410 are newly diagnosed with (2). The surgery and chemotherapy treatment of OC are based on the International Federation of Gynecology and Obstetrics (FIGO) staging (3, 4), but the 5-year relative survival rate (RSR) is below 50%.

For younger women, this means loss of reproductive potential (5). The American Society of Clinical Oncology (ASCO) recommends preserving fertility in women of reproductive age when treating gynecologic tumors other than cervical cancer (6). For example, patients with endometrial cancer (EC) can be treated by oral or intra-uterine progestins with or without hysteroscopic endometrial resection to preserve fertility. In recent years, there were also studies on preoperative biopsy technique and the postoperative recurrence predictor of EC, which increase the possibility of pregnancy in women with preserved fertility (7, 8). Although the National Comprehensive Cancer Network (NCCN) guidelines recommend fertility-sparing surgery (FSS) only for early-stage patients or those with low-risk ovarian tumors (9), these recommendations are based on limited observational evidence (10). One study reported increased provision of FSS in younger vs. older women with no difference in mortality (11). For older women, complete surgical staging may result in decreased quality of life and distress and negatively impact survivorship (12). Therefore, some women without evidence of extra-pelvic disease may consider FSS. Currently, data on OC in women with FSS are limited, while chemotherapy for it is controversial (13). The Surveillance, Epidemiology, and End Results (SEER) database is a cancer database in the United States that has collected information on 34.6% of the American population from 18 different registries. Here, we analyzed the SEER database to determine the risk and prognostic factors of women with FSS, as well as survival outcomes of those with stage I EOC and the effect of chemotherapy. These insights will allow doctors to make better clinical decisions and enable individualized treatment and testing for accurate survival predictions. A nomogram combines multiple variables that may predict and quantify patient survival. Currently, there is no nomogram model for patients with stage I EOC. Therefore, we established a nomogram model for stage I EOC patients with FSS based on findings from the SEER database.

## Materials and Methods

### Data Source and Study Population

**Figure 1:**
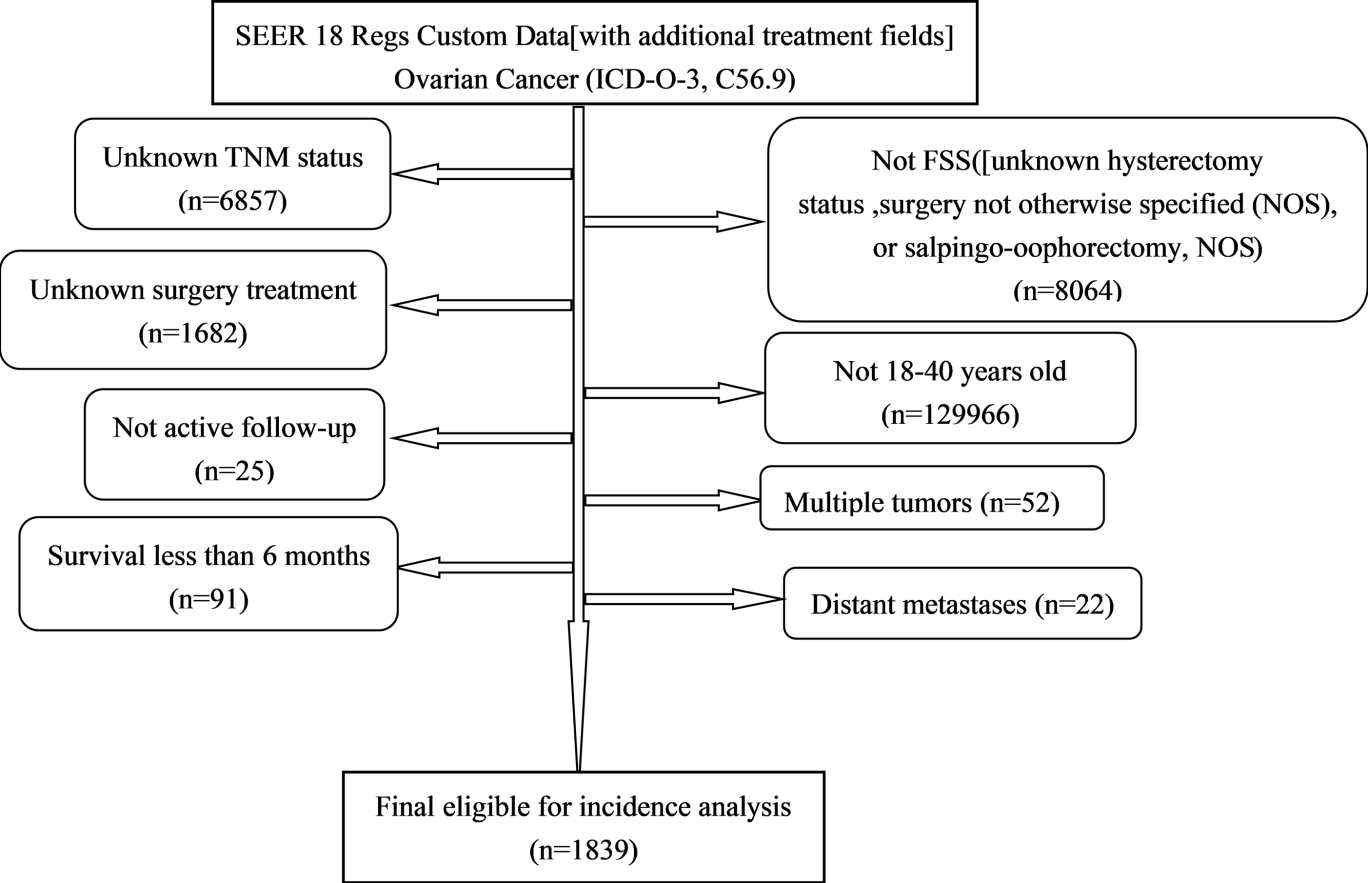
Flowchart of patient selection from the SEER database.

All the primary data were acquired from the SEER database. The SEER∗Stat version 8.3.9 (https://seer.cancer.gov/seerstat/) was used to screen eligible patients who were 18–40 years old with histologically confirmed primary OC (ICD-O-3, C56.9) between 1998 and 2016. We gathered the following information: age at diagnosis, race, grade, historic stage, tumor-node-metastasis (TNM) stage, FIGO stage, marital status [other (separated, divorced, widowed)], surgery treatment (unilateral salpingo-oophorectomy and uterus-sparing), chemotherapy, pathological subtype [Third Edition (ICD-O-3) morphology codes (8441/3, 8442/3, 8460/3, 8461/3, 8462/3, 9014/3 for serous; 8380/3, 8381/3, 8382/3 for endometrioid; 8005/3, 8310/3 for clear cell; 8470/3, 8471/3, 8472/3, 8480/3, 8482/3 for mucinous)], vital status, and survival time. Patients diagnosed by autopsy or death certificate, had multiple tumors or distant metastases, and with a follow-up shorter than 6 months, were excluded from the study. A total of 1,839 patients were eligible for incidence analysis. The primary endpoint was cancer-specific survival (CSS). CSS was defined as the time interval from OC diagnosis to OC-related death (**Figure 1**).

### Statistical Analysis

Univariate and multivariate logistic regression was conducted to determine clinical characteristics and related factors in women with fertility preservation according to chemotherapy. Odds ratios (OR) and 95% confidence intervals (CI) were reported from the logistic regression. Univariate and multivariate Cox regression analyses were employed to identify independent predictors associated with survival by reporting the hazard ratios (HR) and 95% CI. Nomograms were constructed from the predictive model that includes identified prognostic factors. The predictive accuracies of the constructed nomograms were evaluated using the concordance index (C-index). Calibration was done to assess the consistency between the predicted probability and the actual result. Survival comparisons were made using Kaplan–Meier analysis and log-rank tests.

Statistical analyses were all performed using SPSS (version 22.0, IBM Corporation, USA) and R software (version 3.6.3; www.r-project.org/). A two-sided *p* value <0.05 was considered statistically significant.

## Results

### Patient Characteristics

The demographic and clinical characteristics of the 1,839 patients are shown in **Table 1**. Most of them were 18–30 years old (59.3%), White (72.9%), with localized stage (80.4%), unmarried (57.2%), with non-epithelial histologic type (59.2%), with grade 1 (31.8%), T1 (85.4%), N0 (98.0%), and stage I (84.3%). A total of 628 (34.1%) patients received chemotherapy and 1,211 (65.9%) did not. There was an equal number of White patients in both chemotherapy and non-chemotherapy groups. Women with younger, non-localized stage, unmarried, clear cell and non-epithelial histologic type, grades 2–4, T2–3, N1, M1, and stages II–III were more likely to receive chemotherapy.

**Table 1:** Baseline characteristics of women with FSS.

	Total (N = 1,839)	Non-chemotherapy (N = 1,211)	Chemotherapy (N = 628)	P -value*
**Age**				
18–30	1,091 (59.3)	663 (54.7)	428 (68.2)	**<0.001**
31–40	748 (40.7)	548 (45.3)	200 (31.8)	
**Race**				
White	1,341 (72.9)	885 (73.1)	456 (72.6)	0.963
Black	240 (13.1)	158 (13.1)	82 (13.1)	
Other	258 (14.0)	168 (13.8)	90 (14.3)	
**Historic stage**				
Localized	1,479 (80.4)	1,038 (85.7)	441 (70.2)	**<0.001**
Regional	267 (14.5)	143 (11.8)	124 (19.8)	
Distant	93 (5.1)	30 (2.5)	63 (10.0)	
**Marital status**				
Married	679 (36.9)	498 (41.1)	181 (29.8)	**<0.001**
Unmarried	1,052 (57.2)	632 (52.2)	420 (66.9)	
Other	108 (5.9)	81 (6.7)	27 (4.3)	
**Histology**				
Serous	135 (7.3)	108 (8.9)	27 (4.3)	**<0.001**
Endometrioid	282 (15.3)	201 (16.6)	81 (12.9)	
Clear cell	48 (2.6)	28 (2.3)	20 (3.2)	
Mucinous	286 (15.6)	222 (18.3)	64 (10.2)	
Non-epithelial	1,088 (59.2)	652 (53.9)	436 (69.4)	
**Grade**				
1	585 (31.8)	463 (38.2)	122 (19.4)	**<0.001**
2	401 (21.8)	256 (21.1)	145 (23.1)	
3	357 (19.4)	160 (13.2)	197 (31.4)	
4	97 (5.2)	53 (4.4)	43 (6.8)	
Unknown	400 (21.8)	279 (23.1)	121 (19.3)	
**T**				
T1	1,570 (85.4)	1,074 (88.7)	496 (79.0)	**<0.001**
T2	164 (8.9)	97 (8.0)	67 (10.7)	
T3	105 (5.7)	40 (3.3)	65 (10.3)	
**N**				
N0	1,803 (98.0)	1,200 (99.0)	603 (95.8)	**<0.001**
N1	36 (2.0)	11 (1.0)	25 (4.2)	
**FIGO**				
I	1,551 (84.3)	1,070 (88.4)	481 (76.6)	**<0.001**
II	159 (8.6)	96 (7.9)	63 (10.0)	
III	129 (7.1)	45 (3.7)	84 (13.4)	

*Bold P- value, p<0.05.

### Determinants of Chemotherapy

**Table 2** shows the distributions of patient characteristics according to chemotherapy treatment using univariate and multivariate logistic regression. On the multivariate logistic regression analysis, younger age was associated with higher odds of undergoing chemotherapy (vs. 31–40 years old, OR: 0.737, 95% CI: 0.582–0.932). Whereas compared to married patients, unmarried (OR:1.581, 95% CI: 1.240–2.019) patients were associated with higher odds of undergoing chemotherapy. Additionally, compared to patients with serous histologic type, those with clear cell histologic type (OR: 3.047, 95% CI: 1.405–6.608) and non-epithelial histologic type (OR: 3.103, 95% CI: 1.955–5.084) had higher odds of undergoing chemotherapy. Also, compared to patients with grade 1, those with grade 2 (OR: 2.642, 95% CI: 1.956–3.580), grade 3 (OR: 4.560, 95% CI: 3.371–6.198), or grade 4 (OR: 2.722, 95% CI: 1.690–4.371) had higher odds of chemotherapy. Further, patients with stage II (OR: 1.529, 95% CI: 1.067–2.180) and stage III (OR: 3.765, 95% CI: 2.498–5.750) had higher odds of chemotherapy compared to those with stage I.

**Table 2:** Univariate and multivariate logistic regression for associations between patient characteristics and chemotherapy.

Variable	Univariate		Multivariate	
	OR (95% CI)	*p*-value	OR (95% CI)	*p*-value*
**Age**				
18~30	1 (reference)		1 (reference)	
31~40	0.565 (0.461–0.691)	**<0.001**	0.737 (0.582–0.932)	**0.011**
**Race**				
White	1 (reference)			
Black	1.007 (0.751–1.342)	0.961		
Other	1.040 (0.784–1.372)	0.785		
**Marital status**				
Married	1 (reference)		1 (reference)	
Unmarried	1.828 (1.484–2.259)	**<0.001**	1.581 (1.240–2.019)	**<0.001**
Other	0.917 (0.566–1.447)	0.717	0.925 (0.549–1.516)	0.762
**Histology**				
Serous	1 (reference)			
Endometrioid	1.612 (0.993–2.678)	0.058	1.689 (1.003–2.908)	0.053
Clear cell	2.857 (1.398–5.842)	**0.004**	3.047 (1.405–6.608)	**0.005**
Mucinous	1.153 (0.702–1.934)	0.580	1.409 (0.826–2.452)	0.216
Non-epithelial	2.675 (1.751–4.224)	**<0.001**	3.103 (1.955–5.084)	**<0.001**
**Grade**				
1	1 (reference)		1 (reference)	
2	2.150 (1.618–2.862)	**<0.001**	2.642 (1.956–3.580)	**<0.001**
3	4.673 (3.509–6.251)	**<0.001**	4.560 (3.371–6.198)	**<0.001**
4	3.079 (1.961–4.822)	**<0.001**	2.722 (1.690–4.371)	**<0.001**
**FIGO**				
I	1 (reference)		1 (reference)	
II	1.460 (1.040–2.037)	**0.027**	1.529 (1.067–2.180)	**0.020**
III	4.152 (2.861–6.101)	**<0.001**	3.765 (2.498–5.750)	**<0.001**

*Bold P- value, p<0.05.

### Predictors for Survival

In the univariate analysis, age, marital status, histology type, chemotherapy, grade, and FIGO stage were all associated with survival. The multivariate Cox regression model showed that patients with chemotherapy (HR: 0.351, 95% CI: 0.221–0.558) and non-epithelial histologic type (HR: 0.238, 95% CI: 0.138–0.412) had better outcomes compared with the former. Additionally, poor outcomes were seen in patients who were older (HR: 1.777, 95% CI: 1.196–2.640), separated, divorced, or widowed (HR: 2.344, 95% CI: 1.374–3.999) or had grade 3 (HR: 2.923, 95% CI: 1.707–5.004), grade 4 (HR: 7.065, 95% CI: 3.645–13.696), stage II (HR: 7.098, 95% CI: 4.633–10.874), or stage III (HR: 9.882, 95% CI: 6.120–15.958) compared with the former (**Table 3**).

**Table 3:** Univariate and multivariate Cox regression of cancer-specific survival among OC women with FSS.

Variable	Univariate analysis		Multivariate analysis	
	Hazard ratio (95% CI)	*p*-value	Hazard ratio (95% CI)	*p*-value*
**Age**				
15~30	1 (reference)		1 (reference)	
31~44	2.681 (1.887–3.808)	**<0.001**	1.777 (1.196–2.640)	**0.004**
**Race**				
White	1 (reference)			
Black	0.884 (0.522–1.499)	0.647		
Other	0.992 (0.607–1.620)	0.974		
**Marital status**				
Married	1 (reference)		1 (reference)	
Unmarried	0.955 (0.656–1.390)	0.811	1.299 (0.862–1.957)	0.211
Other	3.555 (1.138–5.909)	**<0.001**	2.344 (1.374–3.999)	**0.002**
**Histology**				
Serous	1 (reference)		1 (reference)	
Endometrioid	0.606 (0.368–0.996)	**0.048**	0.883 (0.529–1.476)	0.636
Clear cell	1.615 (0.862–3.026)	0.135	1.705 (0.881–3.299)	0.113
Mucinous	0.384 (0.223–0.663)	**0.001**	0.801 (0.450–1.425)	0.450
Non-epithelial	0.134 (0.081–0.222)	**<0.001**	0.238 (0.138–0.412)	**<0.001**
**Chemotherapy**				
No	1 (reference)		1 (reference)	
Yes	0.448 (0.290–0.692)	**<0.001**	0.351 (0.221–0.558)	**<0.001**
**Grade**				
1	1 (reference)		1 (reference)	
2	2.124 (1.296–3.479)	**0.003**	1.603 (0.966–2.661)	0.068
3	2.555 (1.539–4.241)	**<0.001**	2.923 (1.707–5.004)	**<0.001**
4	5.689 (3.061–10.574)	**<0.001**	7.065 (3.645–13.696)	**<0.001**
**FIGO**				
I	1 (reference)		1 (reference)	
II	5.015 (3.326–7.560)	**<0.001**	7.098 (4.633–10.874)	**<0.001**
III	9.989 (6.487–15.380)	**<0.001**	9.882 (6.120–15.958)	**<0.001**

*Bold P- value, p<0.05.

### Survival Outcomes in Stage I EOC

**Figure 2:**
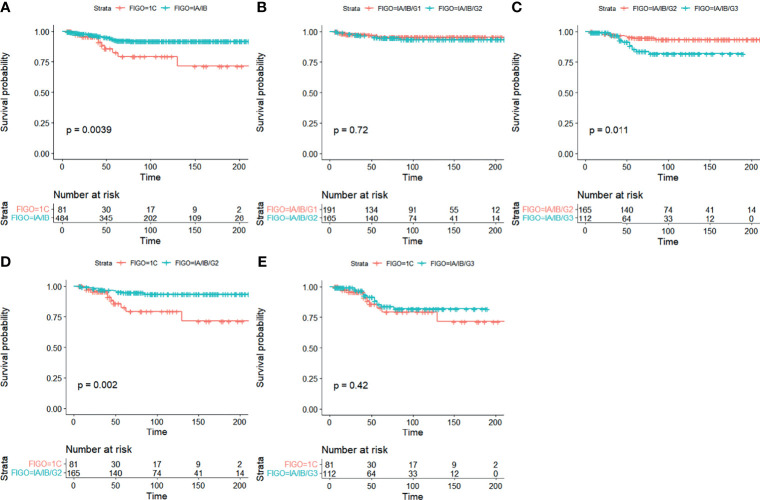
Kaplan–Meier curves for CSS in stage I EOC. **(A)** Stage IA/IB vs. stage IC, **(B)** stage IA/IB-grade 1 vs. stage IA/IB-grade 2, **(C)** stage IA/IB-grade 2 vs. stage IA/IB-grade 3, **(D)** stage IA/IB-grade 2 vs. stage IC, and **(E)** stage IA/IB-grade 3 vs. stage IC.

Analysis of Kaplan–Meier curves in stage I EOC indicated that significant differences were seen in CSS between stage IA/IB and stage IC (5-year CSS 92.8% vs. 80.6%) (**Figure 2A**). Similarly, significant differences were detected in CSS between stage IA/IB-grade 2 and stage IA/IB-grade 3 (5-year CSS 94.7% vs. 85.3%) (**Figure 2C**). Significant differences were seen in CSS between stage IA/IB-grade 2 and stage IC (5-year CSS 94.7% vs. 80.6%) (**Figure 2D**). However, no significant differences were detected in CSS between stage IA/IB-grade 1 and stage IA/IB-grade 2 (**Figure 2B**). Moreover, no significant statistical differences were in CSS between stage IA/IB-grade 3 and stage IC (**Figure 2E**).

**Figure 3:**
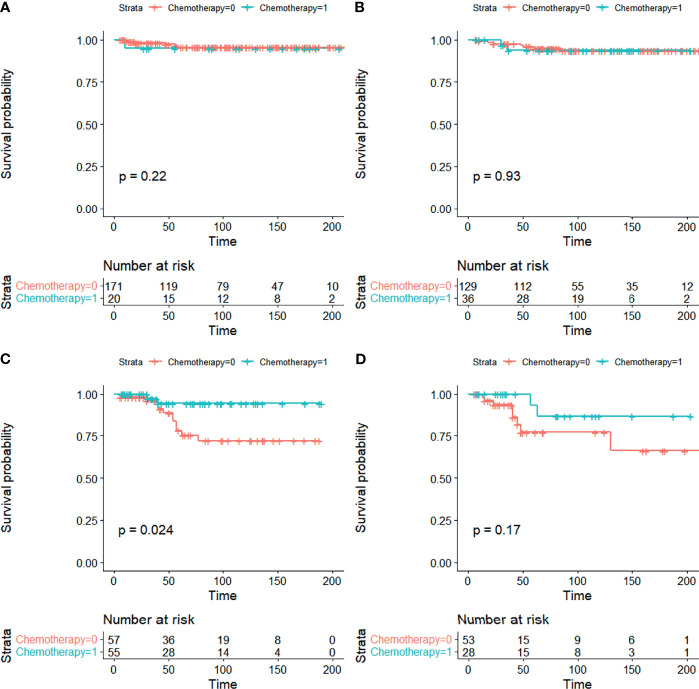
CSS curves stratified in stage I EOC by chemotherapy. **(A)** Stage IA/IB-grade 1, **(B)** stage IA/IB-grade 2, **(C)** stage IA/IB-grade 3, and **(D)** stage IC [0 = non-chemotherapy; 1 = chemotherapy].

Stratification analyses were carried out to control for confounders. CSS curves stratified by chemotherapy are shown in **Figure 3**. In stratification analysis according to FIGO stage and grade, significant differences were seen in CSS when patients were stage IA/IB-grade 3 (5-year CSS 78.1% vs. 94.6%), but not when they were stage IA/IB-grade 1, stage IA/IB-grade 2, or stage IC between non-chemotherapy and chemotherapy groups in EOC (**Figures 3A–D**).

### Construction of a Nomogram Model of CSS for Stage I EOC

**Figure 4:**
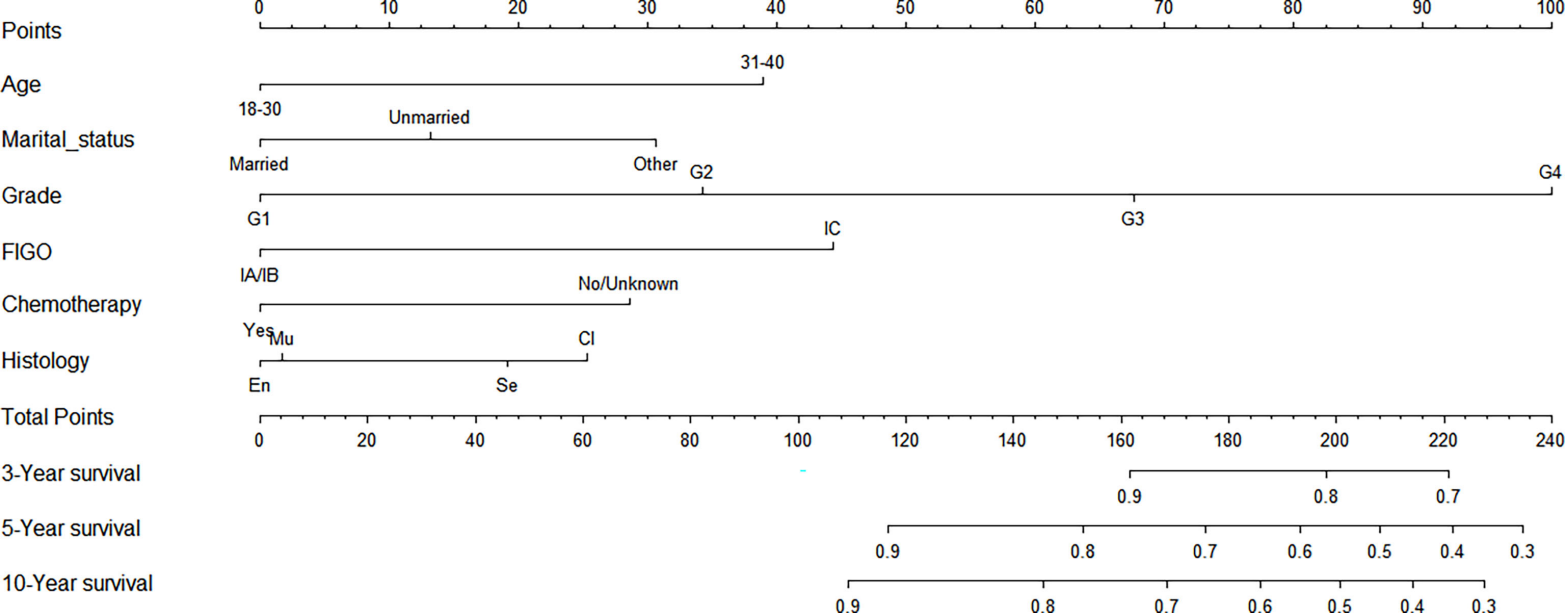
Nomograms to predict 3-, 5-, and 10-year CSS for stage I EOC.

We made a nomogram model of CSS by significant factors among patients of stage I EOC. Each variable could be evaluated with a score from 0 to 100, and the corresponding sum of these scores ranging from 0 to 240 was also assessed accordingly based on the 3-, 5-, and 10-year survival rates varying from 0.1% to 0.9% (**Figure 4**).

### Calibration Chart Among Patients With Stage I EOC

**Figure 5:**
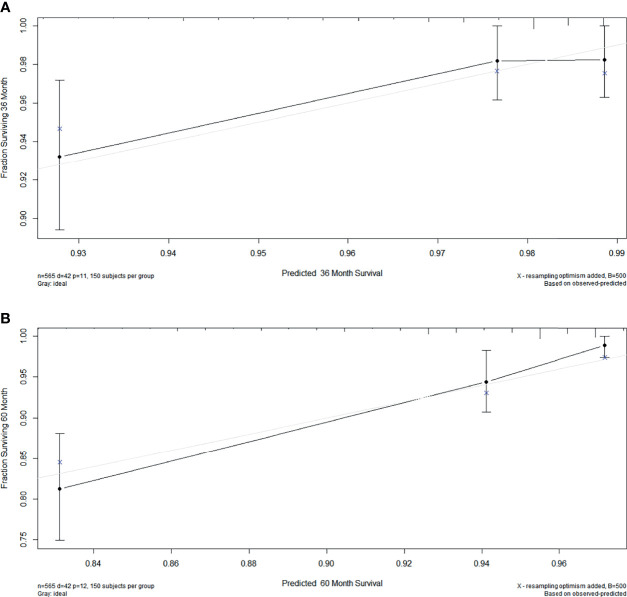
The calibration plot established for the nomogram among patients of stage I EOC. The x-axis describes nomogram-predicted survival; the y-axis indicates observation survival. The graph along the 45° line shows the ideal calibration model, where the predicted probability is consistent with the actual result. **(A)** 3- year, **(B)** 5- year.

The C-index was 0.771 among women with stage I EOC. To further evaluate the consistency of the nomogram, we drew a calibration plot to describe a favorable prediction for 3-year (A) and 5-year (B) CSS among patients with fertility preservation (**Figure 5**).

## Discussion

OC is one of the most common gynecological malignancies. The increasing number of young patients with OC, and the need to preserve their reproductive function during treatment, is essential. In our study, we report the clinical and prognostic characteristics of FSS of patients with OC. Due to the growing interest in FSS in early-stage EOC, we analyzed the survival outcomes of patients with stage I EOC and the role of chemotherapy in it. From this, a prognostic nomogram for stage I EOC was established to allow clinicians to individualize treatment.

Chemotherapy is often used to treat OC (14, 15), and this study demonstrates that it is an independent prognostic factor for CSS in patients. However, chemotherapy has effects on ovarian function, such as reproductive toxicity (16–18). In this study, the multivariate logistic regression indicated that younger women who are unmarried and have grades 2–4, stages II–III, clear cell, or non-epithelial histologic types had higher odds associated with chemotherapy, which is similar to previous studies (19–21). It is understandable that in younger and unmarried women with OC, having children may not be an urgent concern, so they opt for chemotherapy to prevent tumor recurrence. Moreover, one study indicated no association between chemotherapy and decreased fertility in young patients with EOC (22). Also, chemotherapy was the best choice for women with advanced or high-grade OC to prevent recurrence after FSS. In addition, we found that women with OC with a clear cell and non-epithelial histologic type were more likely to undergo chemotherapy. This may be because non-epithelial OC occurs more often in young women, and FSS in non-epithelial OC is not limited by FIGO stage (23). A retrospective study has shown that OC patients with a clear cell histologic type who received chemotherapy had better disease-free survival (DFS) than those who did not (24). In addition, the multivariate Cox model indicated that older women who are separated, divorced, or widowed, did not undergo chemotherapy, and with epithelial histologic type, grades 3–4, or stages II–III had increased risks of correlation with CSS. As expected, FSS was not effective for advanced EOC. In addition, previously published data reported that high-grade tumors should not be considered for FSS due to increased risk of recurrence (25, 26). Previous studies also reported that married women with cancer might have more support from family members, social services, and insurance than those with another marital status, who were at a significantly higher risk of undertreatment and death from cancer (27). Thus, the decision to pursue FSS should be individualized based on disease characteristics.

The prognosis in patients with EOC is often poor. Large retrospective studies and meta-analyses have found that for stage I EOC, FSS did not appear to compromise DFS or overall survival (OS) compared with radical surgery. Although clear cell histology is associated with an increased risk of poor outcomes, some studies have shown that even among patients with stage I clear cells, FSS does not increase the risk of relapse or shorten survival compared with radical surgery (28–30). At present, there is some controversy about chemotherapy for patients with stage I EOC. One study showed that there was no significant difference in DFS and OS between patients with stage IA and IB based on chemotherapy status. However, in patients with intraoperative tumor capsule rupture, DFS in a chemotherapy group was significantly better than that in a non-chemotherapy group (24). According to the NCCN guidelines, observation is an option for patients with stage I, because it has not been demonstrated that chemotherapy provides clear clinical benefit in patients with survival >90% with surgical treatment alone or in patients with low-risk cancer types (9). Our research showed that the prognosis in patients with stage IA/IB-grade 3 or stage IC was worse than those with stage IA/IB-grade 1 or stage IA/IB-grade 2. In addition, significant differences were seen in CSS when patients were stage IA/IB-grade 3 between non-chemotherapy and chemotherapy groups. Although no significant differences were seen when patients were stage IC, we found that chemotherapy improved the survival of patients to some extent.

In addition, a prognostic nomogram model was established for patients with stage I EOC. The nomogram exhibits excellent performance in the results of the calibration and the C-index. The exact score concerning each factor was represented by an elegant graphical interface; and patients being 30–40 years old, with grade 4, with stage IV, being separated, divorced, or widowed, under non-chemotherapy, and with clear cell histology contributed to high scores.

However, a limitation of this study included insufficient information in the SEER database on regimens and the number of cycles of chemotherapy. Also, the SEER database is a registry in the United States, and potential biases are unavoidable.

In conclusion, chemotherapy was often given to fertility-sparing women who are younger, unmarried, with grades 2–4, or with clear cell and non-epithelial histologic type. In addition, those older-aged, separated, divorced, or widowed, with grades 3–4, or with stages II–III were correlated with increased risks of CSS in OC. Further, this study suggests that in EOC, patients with stage IA/IB-grade 1 or stage IA/IB-grade 2 can be followed up without chemotherapy, while those with stage IA/IB-grade 3 and stage IC may need chemotherapy for improved survival.

## Data Availability Statement

All the primary data were acquired fronm the SEER database (https://seer.cancer.gov/seerstat/.).

## Ethics Statement

The informed consent was not required in this study, because personal identifying was not included in the SEER database.

## Author Contributions

Study concept and design: R-fA. Data acquisition: Y-mH, FF. Data analysis and interpretation: Y-mH, HY. Software: J-tH. Manuscript preparation: Y-mH. Critical revision: R-fA. All authors contributed to the article and approved the submitted version.

## Conflict of Interest

The authors declare that the research was conducted in the absence of any commercial or financial relationships that could be construed as a potential conflict of interest.

## Publisher’s Note

All claims expressed in this article are solely those of the authors and do not necessarily represent those of their affiliated organizations, or those of the publisher, the editors and the reviewers. Any product that may be evaluated in this article, or claim that may be made by its manufacturer, is not guaranteed or endorsed by the publisher.
